# Structural variation of the malaria-associated human glycophorin A-B-E region

**DOI:** 10.1186/s12864-020-06849-8

**Published:** 2020-06-29

**Authors:** Sandra Louzada, Walid Algady, Eleanor Weyell, Luciana W. Zuccherato, Paulina Brajer, Faisal Almalki, Marilia O. Scliar, Michel S. Naslavsky, Guilherme L. Yamamoto, Yeda A. O. Duarte, Maria Rita Passos-Bueno, Mayana Zatz, Fengtang Yang, Edward J. Hollox

**Affiliations:** 1 Wellcome Sanger Institute, Hinxton, Cambridge, UK; 2 Present address: Laboratory of Cytogenomics and Animal Genomics (CAG), Department of Genetics and Biotechnology, University of Trás-os-Montes and Alto Douro (UTAD), Vila Real, Portugal; 3 Present address: BioISI – Biosystems & Integrative Sciences Institute, Faculty of Sciences, University of Lisboa, Lisbon, Portugal; 4 Department of Genetics and Genome Biology, University of Leicester, Leicester, UK; 5 Department of Pathology, Faculty of Medicine, Universidade Federal de Minas Gerais, Belo Horizonte, Brazil; 6 Human Genome and Stem Cell Research Center, Department of Genetics and Evolutionary Biology, Instituto de Biociências, Universidade de São Paulo, São Paulo, Brazil; 7 School of Nursing, Universidade de São Paulo, São Paulo, Brazil

**Keywords:** Structural variation, Copy number variation, Inversion, Immune response, Glycophorin, *GYPA*, *GYPB*, *GYPE*, Erythrocytes, Malaria

## Abstract

**Background:**

Approximately 5% of the human genome shows common structural variation, which is enriched for genes involved in the immune response and cell-cell interactions. A well-established region of extensive structural variation is the glycophorin gene cluster, comprising three tandemly-repeated regions about 120 kb in length and carrying the highly homologous genes *GYPA*, *GYPB* and *GYPE*. Glycophorin A (encoded by *GYPA*) and glycophorin B (encoded by *GYPB*) are glycoproteins present at high levels on the surface of erythrocytes, and they have been suggested to act as decoy receptors for viral pathogens. They are receptors for the invasion of the protist parasite *Plasmodium falciparum,* a causative agent of malaria. A particular complex structural variant, called DUP4, creates a *GYPB-GYPA* fusion gene known to confer resistance to malaria. Many other structural variants exist across the glycophorin gene cluster, and they remain poorly characterised.

**Results:**

Here, we analyse sequences from 3234 diploid genomes from across the world for structural variation at the glycophorin locus, confirming 15 variants in the 1000 Genomes project cohort, discovering 9 new variants, and characterising a selection of these variants using fibre-FISH and breakpoint mapping at the sequence level. We identify variants predicted to create novel fusion genes and a common inversion duplication variant at appreciable frequencies in West Africans. We show that almost all variants can be explained by non-allelic homologous recombination and by comparing the structural variant breakpoints with recombination hotspot maps, confirm the importance of a particular meiotic recombination hotspot on structural variant formation in this region.

**Conclusions:**

We identify and validate large structural variants in the human glycophorin A-B-E gene cluster which may be associated with different clinical aspects of malaria.

## Background

Human genetic variation encompasses single nucleotide variation, short insertion-deletions and structural variation. Structural variation can be further divided into copy number variation, tandem repeat variation, inversions and polymorphic retrotransposons. Structural variation is responsible for much of the differences in DNA sequence between individual human genomes [1–3], yet analysis of the phenotypic importance of structural variation has lagged behind the rapid progress made in studies of single nucleotide variation [4–6] . This is mainly because of technical limitations in detecting, characterising, and genotyping structural variants both directly [7] and indirectly by imputation [8]. However, a combination of new technical approaches using genome sequencing data to detect structural variation and larger datasets allowing more robust imputation of structural variation have begun to show that some structural variants at an appreciable frequency in populations do indeed contribute to clinically-important phenotypes [9, 10].

One example of a potentially clinically-important structural variant is a variant at the human glycophorin gene locus called DUP4, which confers a reduced risk of severe malaria and protection against malarial anemia [11–13]. The glycophorin gene locus consists of three ~ 120 kb tandem repeats sharing ~ 97% identity, each repeat carrying a closely-related glycophorin gene, starting from the centromeric end: glycophorin E (*GYPE*), glycophorin B (*GYPB*) and glycophorin A (*GYPA*) [14, 15]. Large tandem repeats, like the glycophorin locus, are prone to genomic rearrangements, and indeed the DUP4 variant is a complex variant that generates a *GYPB*-*GYPA* fusion gene, with potential somatic variation in fusion gene copy number [11, 12]. This fusion gene is expressed and can be detected on the cell surface as the Dantu blood group [11], and erythrocytes carrying this blood group are known to be resistant to infection by *Plasmodium falciparum* in cell culture [16].

How the DUP4 variant mediates resistance to severe malaria is not fully understood. It is well established that both glycophorin A and glycophorin B are expressed on the surface of human erythrocytes and interact with the EBA-145 receptor and the EBL-1 receptor, respectively, of *P. falciparum* [17]. We might expect that direct disruption of ligand-receptor interactions by a glycophorin B-glycophorin A fusion receptor might be responsible for the protective effect of the DUP4 variant. However, recent data suggest that alteration of receptor-ligand interactions is not important. Instead, it seems likely that DUP4 is associated with more complex alterations in the protein levels at the red blood cell surface resulting in increased red blood cell tension, mediating its protective effect against *P. falciparum* invasion [18]. Given the size of effect of the DUP4 variant in protection against malaria (odds ratio ~ 0.6) and the frequency of the allele (up to 13% in Tanzania), it is clinically potentially very significant, although it appears to be geographically restricted to East Africa [11].

Because of the clinical importance of the DUP4 glycophorin variant, and how it can lead to insights on the mechanisms underlying malaria, it is timely to identify and characterise other structural variants in the glycophorin region. Previously, other structural variants in the glycophorin region have been identified in the 1000 Genomes project samples by using sequence read depth analysis of 1.6 kb bins combined with a Hidden Markov Model approach to identify regions of copy number gain and loss [11]. This built upon identification of extensive CNV in this genomic region by array CGH [19] and indeed by previous analysis of rare MNS (Miltenberger) blood groups, such as M^K^, caused by homozygous deletion of both *GYPA* and *GYPB* [14]. The structural variants that were previously identified were classified as DUP and DEL representing gain and loss of sequence read depth respectively. Although only DUP4 has been found to be robustly associated with clinical malaria phenotypes, it is possible that some of the other structural variants are also protective, but are either rare, recurrent, or both rare and recurrent, making imputation from flanking SNP haplotypes and genetic association with clinical phenotypes challenging.

It is important, therefore, to extend this catalogue of glycophorin structural variants at this locus and robustly characterise their nature and likely effect on the number of full-length and fusion glycophorin genes. In this study we characterise and validate glycophorin structural variants from a larger and geographically diverse set of individuals. To detect copy number changes in the glycophorin genomic region, we use sequence read depth analysis of 3234 diploid genomes from across the world, followed by direct analysis of structural variants using fibre-FISH and breakpoint mapping using paralogue-specific PCR and Sanger sequencing. This will allow future development of robust yet simple PCR-based assays for each structural variant and detailed analysis of the phenotypic consequences of particular structural variants on malaria infection and other traits. We also begin to examine the pattern of distribution of different variants across the world, and the pattern of structural variation breakpoints in relation to their mechanism of generation and known meiotic recombination hotspots within the region. Together, this allows us to gain some insight into the evolutionary context of the extensive structural variation at the glycophorin locus.

## Results

### Structural variation using sequence read depth analysis

Previous work by us and others has shown that unbalanced structural variation - that is, variation that causes a copy number change - can be effectively discovered by measuring the relative depth of sequence reads across the glycophorin region [11, 12] . We analysed a total of 3234 diploid genomes from four datasets spanning the globe - the 1000 Genomes phase 3 project set, the Gambian Genome Variation project, the Simons diversity project, and the Brazilian genomes project. We took a different sequence read depth approach to that previously used, counting the reads that map to the glycophorin repeat region and dividing by the number of reads mapping to a nearby non-structurally variable region to normalise for read depth. By analysing each cohort of diploid genomes as a group, we could identify outliers where a higher value indicated a potential duplication or more complex gain of sequence, and lower values indicated a potential deletion (Supplementary Fig. 1). Sequence read depth was analysed in 5 kb windows across each of the outlying diploid genomes to identify and classify the structural variant.

Since structural variant calling had been previously done on the 1000 Genomes project cohort, this provided a useful comparison to assess our approach. We analysed samples from this cohort and identified five distinct deletions carried by 88 individuals, and 16 distinct duplications carried by 34 individuals (Table 1) that were all previously identified (Supplementary data). We also identified a new duplication variant, termed DUP29 (a duplication of *GYPB*), that had not been identified previously in that cohort. However, as expected, smaller duplications, most notably DUP1, were not detected by our approach. We extended our analysis to Gambian genomes and identified 51 samples with DEL1 or DEL2 variants, and DEL16, subsequently characterized in the Brazilian cohort below. Two samples were heterozygous for the duplication DUP5.

**Table 1:** Glycophorin structural variants identified in this study

Variant	Proximal breakpoint hg19	Distal breakpoint hg19	Variant size (kb)	Resolution of breakpoint (kb)	Index sample	Genes involved	Breakpoint identification method	In ref. [11]
DEL1	chr4:144835143–144,835,279	chr4:144945375–144,945,517	110	0.143	NA19223	*GYPB*	*PCR-Sanger*	*Yes*
DEL2	chr4:144912872–144,913,001	chr4:145016127–145,016,256	103	0.130	NA19144	*GYPB*	*PCR-Sanger*	*Yes*
DEL4	chr4:144750739–144,760,739	chr4:144950739–144,960,739	200	10	HG01986	*GYPB,GYPE*	*1000G Seq*	*Yes*
DEL6	chr4:144780045–144,780,137	chr4:145004120–145,004,212	224	0.093	HG04039	*GYPE* and *GYPB*	*PCR-Sanger*	*Yes*
DEL7	chr4:144780111–144,780,497	chr4:144900945–144,901,334	121	0.390	HG02716	*GYPE*	*PCR-Sanger*	*Yes*
DEL13	chr4:144925739–144,935,739	chr4:145035739–145,045,739	110	10	NA20867	*GYPA/B fusion gene*	*1000G Seq*	*Yes*
DEL15	chr4:144800739 144,802,739	chr4:144920739–144,922,739	119	2	HGDP01172	*GYPB/E fusion gene*	*SD*	*No*
DEL16	chr4:144752739–144,754,739	chr4:144952739–144,954,739	200	2	BR1296010301	*GYPE* and *GYPB*	*SD*	*No*
DEL17	chr4:144882739–144,987,739	chr4:144984739--144,987,739	103	3	BR1183605501	*GYPB*	*SD*	*No*
DEL18	chr4:144755739–144,757,739	chr4:144875739–144,878,739	123	2	BR1099223302	*GYPE*	*SD*	*No*
DUP2	chr4: 145039739–145,041,739	chr4: 144919739–144,921,739	120	2	NA18593	*GYPB/A fusion gene*	*PCR-Sanger*	*Yes*
DUP3	chr4:145004465–145,004,526	chr4:144780388–144,780,449	224	0.062	NA19360	*GYPB, GYPE*	*PCR-Sanger*	*Yes*
DUP4	Multiple	Multiple	n/a	n/a	HG02554	*GYPB/A fusion gene, GYPE*	*Ref.* [11]	*Yes*
DUP5	Multiple, including chr4:145113700	Multiple, including chr4:144936865	n/a	0.001	HG02585	*GYPB, GYPE*	*PCR-Sanger*	*Yes*
DUP7	chr4:144895000–144,905,000	chr4:144775000–144,785,000	120	10	HG02679	*GYPE*	*1000G Seq*	*Yes*
DUP8	chr4:14504573 9–145,048,739	chr4:144808739–144,810,739	240	3	I1_S_Irula1, HG03837	*GYPB, GYPE/A fusion gene*	*SD*	*Yes*
DUP14	chr4:144853613–144,853,688	chr4:144723019–144,723,094	131	0.076	NA18646	*GYPE*	*PCR-Sanger*	*Yes*
DUP22	chr4:144926739–144,929,739	chr4:144881739–144,884,739	45	3	BR210800138, HG02181	*GYPB (partial)*	*SD*	*Yes*
DUP26	chr4:145065739–145,075,739	chr4:144830739–144,840,739	155	10	HG03729	*GYPA*	*1000G Seq*	*Yes*
DUP27	chr4: 145039739–145,041,739	chr4: 144919739–144,921,739	120	2	NA12249	*GYPB/A fusion gene*	*PCR-Sanger*	*Yes*
DUP29	chr4:144939393–144,939,452	chr4:144825584–144,825,643	114	0.060	HG03686	*GYPE* and *GYPB*	*PCR-Sanger*	*No*
DUP30	chr4:144989739–144,991,739	chr4:144885739–144,887,739	102	2	HGDP00543	*GYPB*	*SD*	*No*
DUP33	chr4:144959739–144,962,739	chr4:144849739–144,851,739	111	3	BR54409051	*GYPB*	*SD*	*No*
DUP34	chr4:145002739–145,004,739	chr4:144900739–144,902,739	102	2	BR1086675791	*GYPB*	*SD*	*No*
DUP35	chr4:144878739–144,880,739	chr4:144758739–14,476,073,939	120	2	BR981404021	*GYPE*	*SD*	*No*

Notes: SD = sequence depth analysis of high coverage genomic sequencing. DUP19 (NA19223), DUP25 (HG02031), DUP28 (NA19084) no clear 5 kb window pattern, DEL4 and DEL16, and DUP2 and DUP27 share overlapping breakpoint regions and may be the same variants. DUP23 (HG02491) and DUP24 (hg03837), identified by reference [11], share population and breakpoint regions with DUP8 and are classified as DUP8. The column titled “in ref. [11]” indicates whether the variant was previously observed by Leffler et al. (reference [11])

Both 1000 Genomes and Gambian Genome Variation samples have been sequenced to low depth. High depth sequencing will allow more robust identification of structural variation by improving the signal/noise ratio of sequence read depth analysis. We analysed the publically available high-depth data from the Simons Diversity Project for glycophorin variation. From the 273 individuals, 4 different deletion types were carried by 13 individuals, and 3 different duplication types were carried by 5 individuals. A novel deletion, DEL15 was identified which deleted part of *GYPB* and part of *GYPE* in an individual from Bergamo in Italy, and a novel duplication was observed in three individuals from Papua New Guinea, termed DUP30 and duplicating the *GYPB* gene. Another duplication variant, DUP8, is the largest variant found so far. It involves a duplication involving two glycophorin repeat units, 240 kb in total, and creates an extra full length *GYPB* gene and a *GYPE-GYPA* fusion gene (Table 1).

Further samples sequenced to high coverage diploid genomes from Brazil were analysed, which, given the extensive admixture from Africa in the Brazilian population, are likely to be enriched for glycophorin variants from Africa. Three new duplication variants (DUP33-DUP35) and three new deletion variants were found (DEL16, DEL17, DEL18), two of which of which delete the *GYPB* gene (Table 1).

### Fibre-FISH analysis of structural variants

**Fig. 1:**
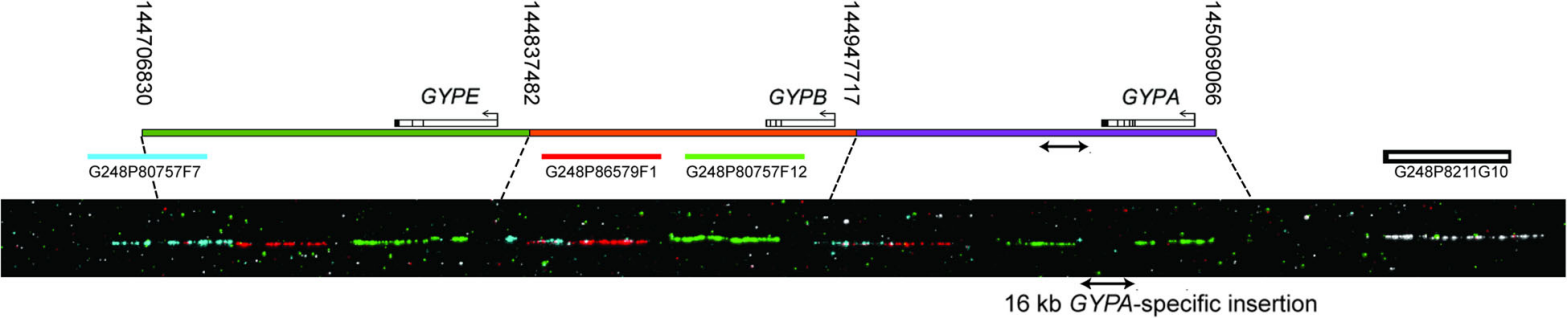
Structure of the glycophorin reference allele. A representation of the reference allele assembled in the GRCh37/hg19 assembly is shown, with the three distinct paralogous ~ 120 kb repeats of the glycophorin region coloured green, orange and purple, carrying *GYPE*, *GYPB* and *GYPA* respectively. Numbers over the start and end of each paralogue represent coordinates in chromosome 4 GRCh37/hg19 assembly. Coloured bars represent fosmids used as probes in fibre-FISH, with the fosmid identification number underneath. The lower black panel is an example fibre FISH image of this reference haplotype (from sample HG02585). The fibre-FISH image is scaled approximately to match the reference above it, with approximate boundaries between glycophorin repeats shown as dashed lines

Sequence read depth analysis shows copy number gain and loss with respect to the reference genome to which the sequence reads are mapped, but it does not determine the physical structure of the structural variant. For all glycophorin structural variants we identified in the 1000 Genomes samples (with the exception of the smaller DUP22), matched lymphoblastoid cell lines were available allowing us to use fibre-FISH in order to determine the physical structure of these variants. In all cases, a set of multiplex FISH probes, with each probe being visualised with a unique fluorochrome, was used so that the orientation and placement of the repeats could be identified (Fig. 1). The repeated nature of the glycophorin region means that the green and red probes from the *GYPB* repeat cross-hybridise with the other repeats, with the *GYPA* repeat is distinguishable from the *GYPB* and *GYPE* repeats by a 16 kb insertion resulting in a small gap of signal in the green probe (Fig. 1).

**Fig. 2:**
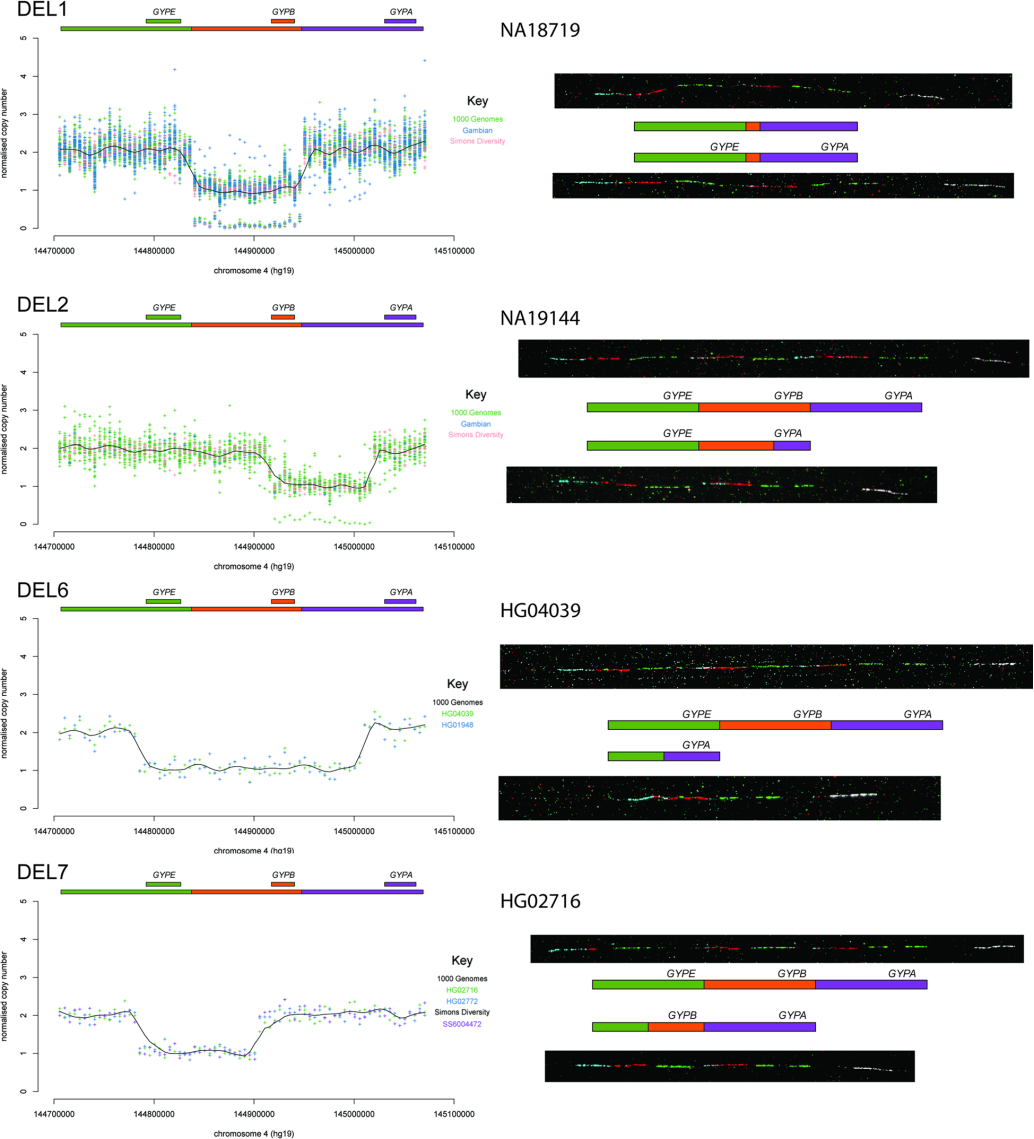
Fibre-FISH validation of four glycophorin deletions. Sequence read depth (SRD) analysis of selected deletions (DEL1, DEL2, DEL6, DEL7) is shown on the left. The sequence read depth for each 5 kb window is shown as a point coloured according to the key on each plot either by sample or by cohort. The solid black line is the Loess best-fit line through the points. Individuals homozygous or DEL1 or DEL2, are shown in the plot with a very low sequence read depth (~ 0) across the deleted region. Above each plot the coloured bars show the glycophorin repeat regions, as in Fig. 1. The smaller coloured bars represent the location of each glycophorin gene (*GYPE, GYPB, GYPA*) labelled above each one. Representative fibre-FISH images from the index sample of each variant are shown on the right, with clones and fluorescent labels as shown in Fig. 1. All index samples apart from NA18719 are heterozygous, with a representative reference (top) and variant (bottom) allele from that sample shown. A schematic diagram next to the corresponding fibre-FISH image shows the structure of each allele inferred from the fibre-FISH and SRD analysis

**Fig. 3:**
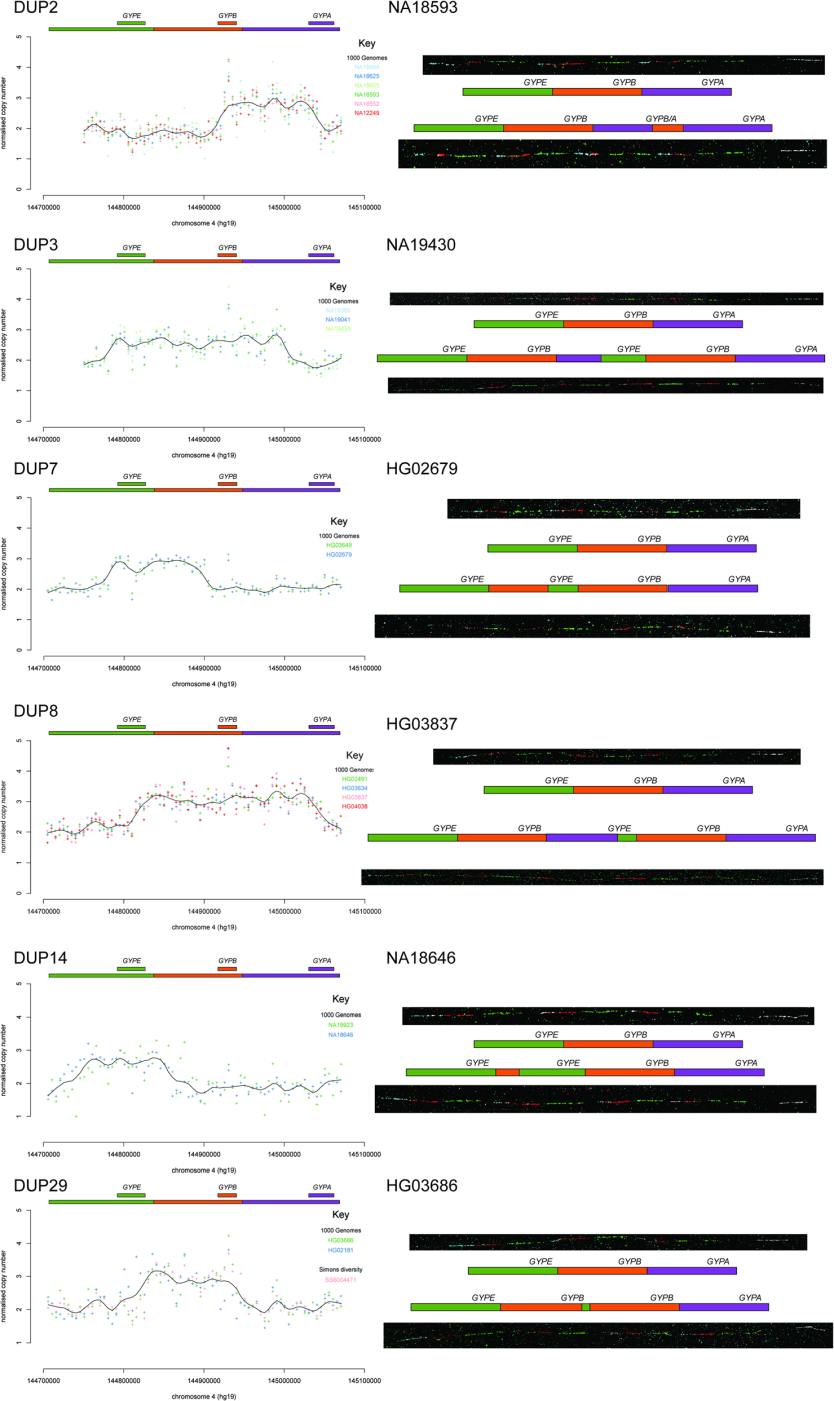
Fibre-FISH validation of six glycophorin duplications. Sequence read depth (SRD) analysis of selected duplications (DUP2, DUP3, DUP7, DUP8, DUP14 and DUP29) is shown on the left. The sequence read depth for each 5 kb window is shown as a point coloured according to the key on each plot either by sample or by cohort. The solid black line is the Loess best-fit line through the points. Above each plot the coloured bars show the glycophorin repeat regions, as in Fig. 1. The smaller coloured bars represent the location of each glycophorin gene (*GYPE, GYPB, GYPA*) labelled above each one. Representative fibre-FISH images from the index sample of each variant are shown on the right, with clones and fluorescent labels as shown in Fig. 1, with an additional green-labelled PCR product specific to the glycophorin E repeat for HG03686. All index samples are heterozygous, with a representative reference and variant allele from that sample shown. A schematic diagram next to the corresponding fibre-FISH image shows the structure of each allele inferred from the fibre-FISH and SRD analysis

For most variants the fibre-FISH results confirmed the structure previously predicted [11] and expected if the variants had been generated by non-allelic homologous recombination (NAHR) between the glycophorin repeats (Figs. 2 and 3). However, three variants showed a complex structure that could not be easily predicted from the sequence read depth analysis. The DUP4 variant shows a complex structure and has been described previously [12] . Two other structural variants (DUP5 and DUP26) also showed complex patterns of gains or losses, and fibre-FISH clearly shows the physical structure of the variant, including inversions.

**Fig. 4:**
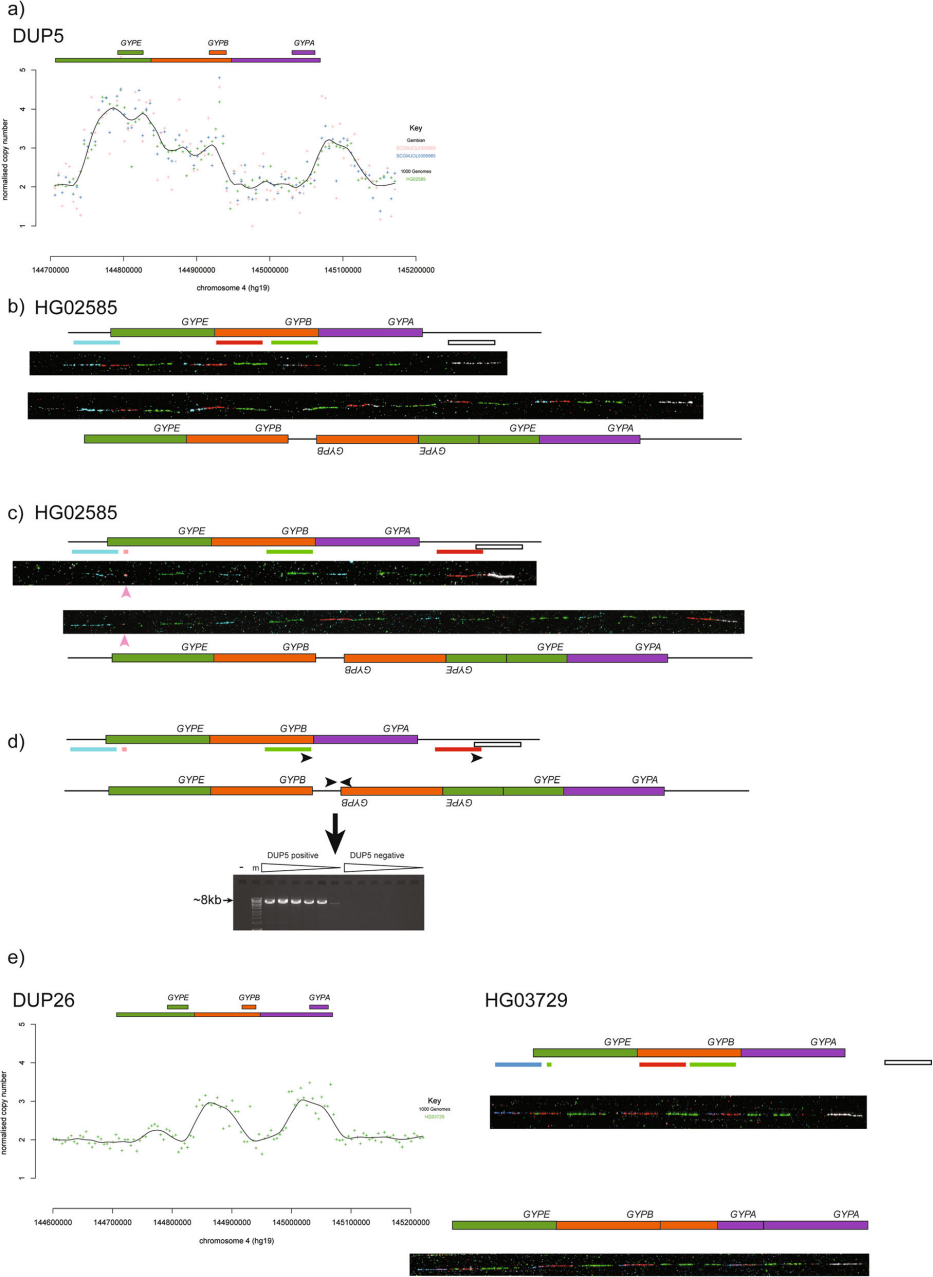
Analysis of DUP5 and DUP26 complex structures. **a** Sequence read depth (SRD) analysis of three individuals heterozygous for the DUP5 variant. **b** Representative fibre-FISH images from the DUP5 index sample HG02585. Clones and fluorescent labels as shown in Fig. 1. **c** Representative fibre-FISH images from the DUP5 index sample HG02585. Clones and fluorescent labels as shown in Fig. 1, except the red probe is fosmid G248P89366H1 and the pink probe is the glycophorin E repeat-specific PCR product. **d** Schematic showing design of PCR primers for specific amplification (black arrows) on reference and DUP5 structures. The ethidium bromide stained agarose gel shows a ~ 8 kb PCR product generated by these DUP5 specific primers. HG02554 is the DUP5 sample, “-” indicates a negative control with no genomic DNA and the marker, indicated by “m”, is Bioline Hyperladder 1 kb+. The triangles indicate increasing PCR annealing temperature from 65 °C to 67 °C. **e** Sequence read depth (SRD) analysis (left) and fibre-FISH analysis (right) of the index sample HG03729 heterozygous for DUP26 variant. Fosmid clones for fibre-FISH are as Fig. 1, except with the addition of the glycophorin E repeat-specific PCR product labelled in pink (**c**, **d**) or green (**e**)

The more frequent of these two complex structural variants, DUP5, seems to be restricted to Gambia, as it is found once in the GWD population from the 1000 Genomes project and twice in the Jola population from the Gambian Genome Variation project (Table 1). Sequence read depth analysis suggests that DUP5 has two extra copies of *GYPE* and an extra copy of *GYPB*, with an additional duplication distal of *GYPA* outside the glycophorin repeated region (Fig. 4a). Fibre-FISH analysis on cells from an individual carrying the DUP5 variant (HG02585) confirmed heterozygosity of the variant, with one allele being the reference allele, and revealed, for the first time, that the variant allele presents a complex pattern of duplication and rearrangement, with part of the fosmid (pseudocoloured in white) mapping distal to *GYPA* being translocated into the glycophorin repeated region, adjacent to the green-coloured fosmid (Fig. 4b). Alternative fibre-FISH analysis using an additional fosmid probe mapping distally, and labelled in red, confirmed this (Fig. 4c). The pattern of FISH signals occurring distally to the translocation suggests that the immediately adjacent glycophorin repeat is inverted. To distinguish the distal end of the *GYPB* repeat from the distal end of the *GYPE* repeat, a pink-coloured probe from a short *GYPE*-repeat-specific PCR product was also used for fibre-FISH, and clearly shows only a single copy of the distal end of the *GYPB* repeat in the DUP5 variant, at the same position as the reference. The predicted breakpoint between the non-duplicated sequence distal to *GYPA* and duplicated sequence within the duplicated region was amplified by PCR and Sanger sequenced, confirming that the non-duplicated sequence was fused to an inverted *GYPB* repeat sequence (Fig. 4d). The model suggested by the fibre-FISH and breakpoint analysis is consistent with the overall pattern of sequence depth changes observed (Fig. 4a). The sequence outside the glycophorin repeat corresponds to an ERV-MaLR long terminal retroviral element, but the sequence inside the glycophorin repeat sequence is not, suggesting that non-allelic homologous recombination was not the mechanism for formation of this breakpoint. However, there is a 4 bp microhomology (GTGT) between the two sequences, suggesting that microhomology-mediated end joining could be a mechanism for formation of this variant.

The DUP26 variant was observed once, in sample HG03729, an Indian Telugu individual from the United Kingdom, sequenced as part of the 1000 Genomes project. Sequence read depth analysis predicts an extra copy of the glycophorin repeat, partly derived from the *GYPB* repeat and partly from the *GYPA* repeat (Fig. 4e). The fibre-FISH shows an extra repeat element that is *GYPB*-like at the proximal end and *GYPA*-like at the distal end, and carries the *GYPA* gene. This structure is unlikely to have been generated by a straightforward single NAHR event, and we were unable to resolve the breakpoint at high resolution.

### Breakpoint analysis of structural variants

**Fig. 5:**
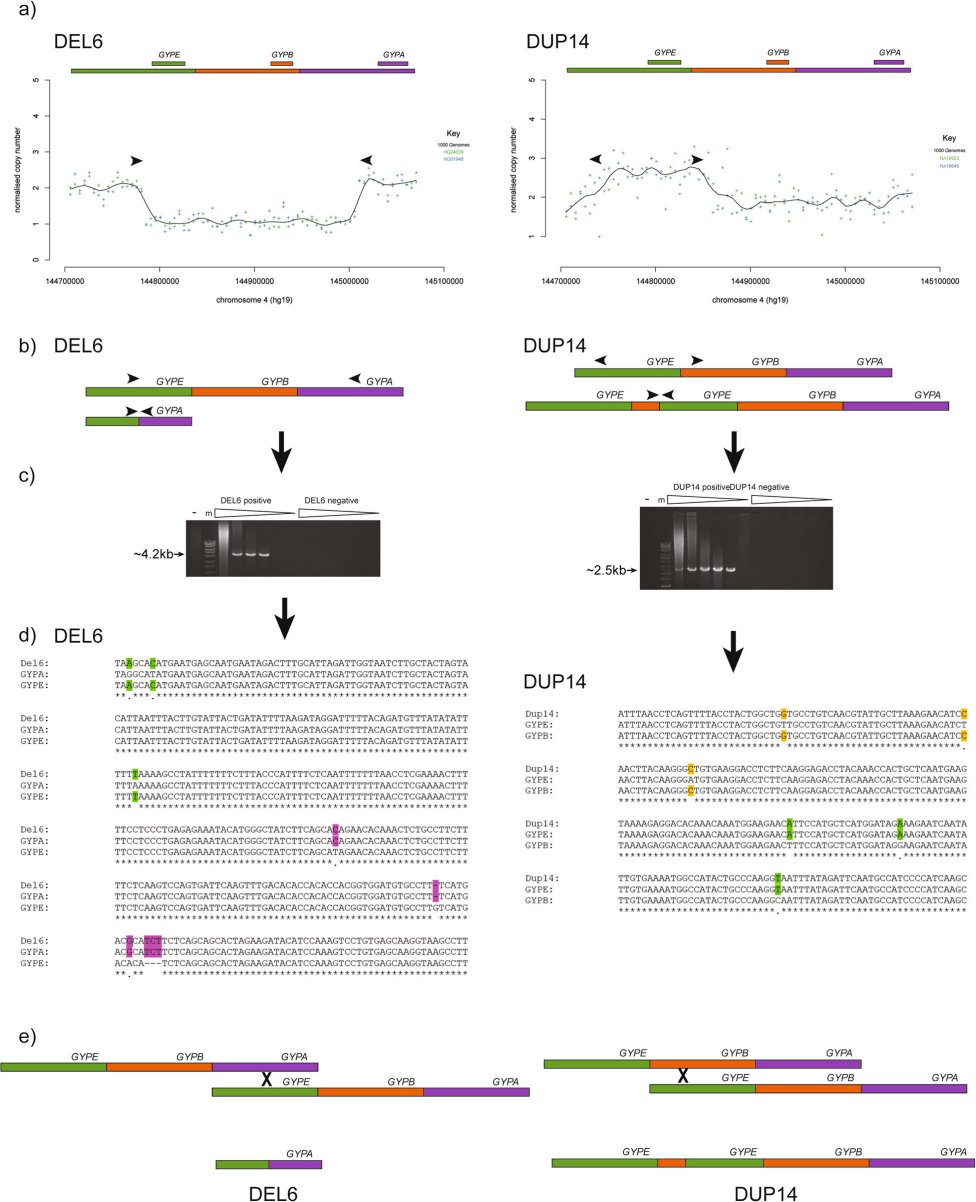
Examples of refining breakpoints of a deletion (DEL6) and a duplication (DUP14). **a** Sequence read depth analysis, indicating position of PCR primers (not to scale). **b** Variant model, showing position of primers on reference and variant. **c** Agarose electrophoresis of long PCR products using variant-specific primers indicated in b). “-” indicates a negative control with no genomic DNA and the marker, indicated by “m”, is Bioline Hyperladder 1 kb+. The triangles indicate increasing PCR annealing temperature from 58 °C to 67 °C. **d** Multiple sequence alignment of the variant-specific PCR product, with homologous sequence on the *GYPA* repeat and the *GYPE* repeat. *GYPE*-specific variants are in green, *GYPA*-repeat-specific variants are in purple. **e** A model of the generation of the variants by NAHR

Defining the precise breakpoint of the variants can allow a more accurate prediction of potential phenotypic effects of each variant by assessing, for example, whether a glycophorin fusion gene is formed or whether key regulatory sequences are deleted. We used two approaches to define breakpoints. The first approach identified the two 5 kb windows that spanned the change in sequence read depth at both ends of the deletion or duplication, and by designing PCR primers to specifically amplify across the junction fragment (Fig. 5a, b), variant-specific PCR amplification produces an amplicon that can be sequenced (Fig. 5c). After Sanger sequencing the amplicons, the breakpoint can be shown to be where a switch occurs between paralogous sequence variants (PSVs) that map to different glycophorin repeats (Fig. 5d), supporting the model that a NAHR mechanism is responsible for generating these structural variants (Fig. 5e). The second approach makes use of high depth sequencing. The two 5 kb windows spanning the change in sequence read depth are again identified and sequence read depth calculated in 1 kb windows to further refine the breakpoint. The sequence alignment spanning the two 1 kb windows is examined manually for paired sequence reads where the gap between the aligned pairs is consistent with the size of the variant, or where both sequence pairs align but one aligns with multiple sequence mismatches.

With the exception of DEL4, DUP7 and DUP26, where only low-coverage sequence was available, all other breakpoints could be localised to between 10 kb and 1 bp. For most variants, the breakpoints occur between genes resulting in loss or gain of whole genes, and therefore likely to show gene dosage effect. It is known that DUP4 results in a *GYPB*-*GYPA* fusion gene that codes for the Dantu blood group, and a fusion gene is also predicted for DUP2, DUP8 and DEL15. The DUP2 variant generates a *GYPB*-*GYPA* fusion gene comprising exons 1–2 of *GYPB* and exons 4–7 of *GYPA* corresponding to the St^a^ (GP.Sch) blood group [20]. Breakpoint analysis of NA12249, the sample carrying the DUP27 variant, showed that DUP27 breakpoint is in the same intron as DUP2 (Supplementary Fig. 2). By using a variant-specific PCR primer pair (Supplementary Table 1) followed by Sanger sequencing, we show the exact breakpoint is complex, as the *GYPA*-like sequence does not show a simple switch to *GYPB*-like sequence but rather shows a pattern of alternate patches of *GYPB-* and *GYPA*-like sequence, suggesting a history of gene conversion events between the glycophorin repeat regions (Supplementary Fig. 2). Although the variants are the same across most of the sequence, two variants are *GYPB*-like in DUP2 and *GYPA*-like in DUP27. This can be explained either by two different recombination events generating the DUP2 and DUP27 variants, or their being exactly the same variant, generated by the same recombinational event, but distinguished by a later gene conversion event on DUP27. At present, therefore, it is unclear whether DUP27 is exactly the same variant as DUP2, and sequencing of more examples of both variants is needed.

The DUP8 variant is predicted to generate a fusion gene consisting of exon 1 of *GYPE* and exons 2–7 of *GYPA*, and the DEL15 variant is predicted to combine the first two exons of *GYPB* with the final three exons of *GYPE*. It is unlikely that DUP8 has a phenotype, given the involvement of the 5′ end of *GYPE*, which is not expressed. The DEL15 variant is predicted to generate a *GYPB*-*GYPE* peptide, and the breakpoint between exon 1 of *GYPB* and exon 2 of *GYPE* is consistent with the variant that causes the rare U- blood group phenotype, resulting in a lack of expression of glycophorin B in homozygotes [21, 22]. It has been shown that the U- blood group can also be caused by the more common DEL1 and DEL2 alleles, both of which also result in *GYPB* deletion [23]. Other variants involve breakpoints within 1 kb of a gene coding region and could potentially affect expression levels of the neighbouring gene.

### Mechanism of formation of structural variants

**Fig. 6:**
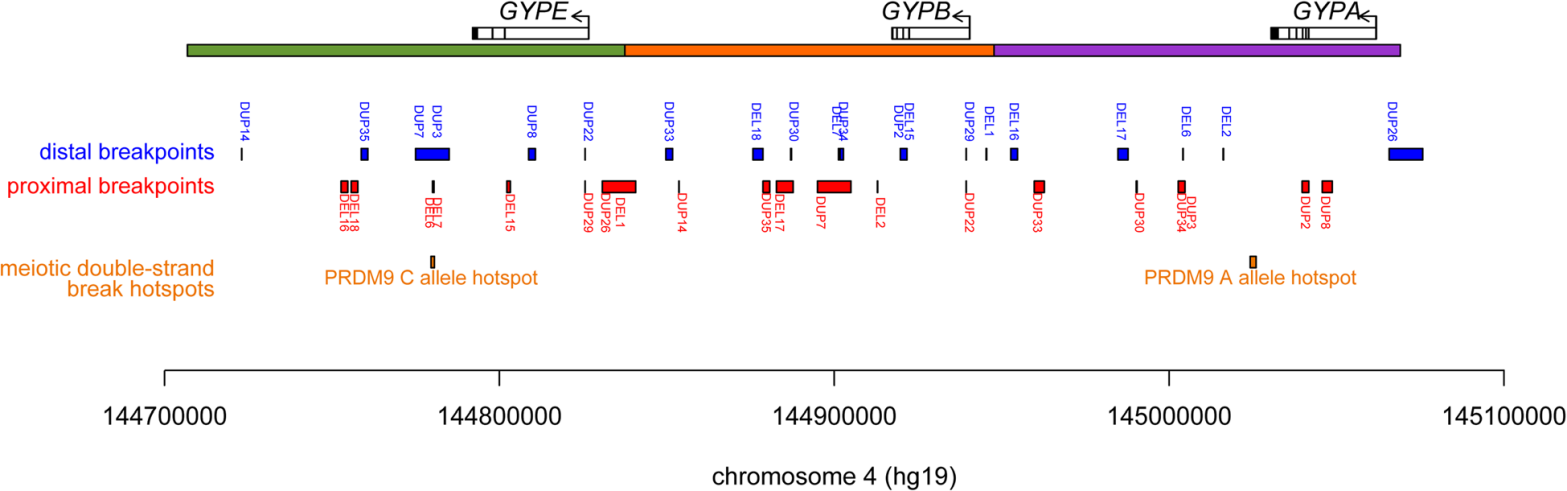
Structural variant breakpoints and meiotic recombination hotspots. The glycophorin region is shown together with the glycophorin genes. Below are the breakpoint regions for each structural variant, labelled in blue for the distal breakpoint in the variant, and red for the proximal breakpoint in the variant. Meiotic double strand break hotspots, corresponding to recombination hotspots [25] are shown in orange, labelled the PRDM9 allele responsible for activating that hotspot

The pattern of deletions and duplications observed is consistent with a simple NAHR mechanism of formation for the variants (Fig. 5e), with the exception of DUP5 and DUP26. We investigated whether the breakpoints we had found co-localised with known meiotic recombination hotspots previously determined by anti-DMC1 ChIP-Seq of the testes of five males [24] . Importantly, the recombination hotspot dataset mapped hotspots in individuals carrying different alleles of the highly-variable PRDM9 protein, a key determinant of recombination hotspot activity, with different alleles activating different recombination hotspots. The glycophorin region contains one hotspot regulated by the PRDM9 A allele, common in Europeans (allele frequency 0.84), and the PRDM9 C allele, common in sub-Saharan Africans (allele frequency 0.13). In our data we found no breakpoints coincident with the PRDM9 A allele hotspot but 4 breakpoints coincident with the PRDM9 C allele hotspot (Fig. 6), as observed previously [11]. The overlap between the PRDM9 C allele hotspot and the structural variant breakpoints is statistically significant (two-tailed Fisher’s exact test, *p* = 0.012) and reflects the observation that there are more different rare structural variants in sub-Saharan African populations, with high frequencies of the C allele, than in European populations where the C allele is almost absent (allele frequency 0.01) [26]. Consistent with this, the four variants where breakpoints span the PRDM9 C allele hotspot (DUP3, DUP7, DEL6, DEL7) are found in the sub-Saharan African or Admixed American populations (Table 2).

**Table 2:** Global distributionof glycophorin structural variants

Continental grouping	1000 Genomes					Gambian	Simons	Brazilian
	EUR	AFR	SAS	EAS	AMR	AFR	ALL	AMR
Total number of chromosomes	600	640	386	606	258	782	546	2650
DEL1	0	53	0	1	1	55	7	19
DEL2	0	26	0	0	2	2	4	12
DEL4/16	0	1	0	0	0	1	0	3
DEL6	0	0	1	0	1	0	0	0
DEL7	0	2	0	0	0	0	1	0
DUP2/27	0	1	1	11	1	0	0	7
DUP3	0	4	0	0	0	0	0	0
DUP5	0	1	0	0	0	2	0	0
DUP7	0	1	0	0	1	0	0	2
DUP8	0	0	4	0	0	0	1	2
DUP29	0	0	1	0	0	0	1	0
DUP22	0	0	0	1	0	0	1	1
DUP30	0	0	0	0	0	0	3	0
DUP35	0	0	0	0	0	0	0	2

Notes: Variants observed more than once are included. The full list of individuals with different glycophorin variants, together with their population of origin, is available as supplementary data

## Discussion

We have characterised a number of structural variants at the human glycophorin locus (Supplementary Fig. 3). These are almost always large deletions or duplications involving the loss or gain of one or occasionally two glycophorin repeat regions of about 120 kb. These losses and gains are consistent with an origin by non-allelic homologous recombination (NAHR) between glycophorin repeats, with particular involvement of the PRDM9 C allele, which is at appreciable frequencies in African populations and directs high recombination rates at its cognate recombination hotspots. A more complex variant, termed DUP5, was also characterised, and was shown to be an inversion-duplication with a breakpoint suggesting generation by at least one microhomology-mediated end-joining event involving DNA sequence outside the glycophorin repeat region. Similarly, DUP26 is a complex variant that is unlikely to have been generated by a single NAHR event.

Only DEL1, DEL2 and DUP2 are frequent variants. Both DEL1 and DEL2 delete the *GYPB* gene and it is tempting to speculate that their high frequency in African populations and populations with African admixture is due to selection. Indeed, erythrocytes from individuals showing the U- blood group and therefore homozygous for *GYPB* deletion are resistant to *P. falciparum* infection in vitro [16, 23, 27]. However, the absence of epidemiological evidence for any protective effect against malaria argues that malaria is not the cause of this selection, so this remains speculation. DUP2 is at notable frequencies particularly in East Asia, and is predicted to generate a *GYPB*-*GYPA* fusion gene corresponding to the St^a^ blood group, which is known to be at appreciable frequencies in East Asia [28, 29]. In this region, malaria infections are caused by *Plasmodium falciparum* as well as *Plasmodium vivax*; alternatively, this fusion gene may facilitate glycophorins acting as a decoy receptor for other pathogens, such as hepatitis A virus [30, 31]. Previous work has shown that DUP2 has arisen on multiple haplotype backgrounds [11], which suggests a large East Asian population panel is need for future accurate imputation.

Other variants seem either to be geographically localised (for example DUP5) or very rare and detected as singletons in our dataset. This suggests that analysis of other large genomic datasets will discover further unique glycophorin structural variants, and that much glycophorin structural variation is individually rare but collectively more frequent, leading to challenges in imputing glycophorin structural variation from SNP GWAS data.

In contrast to other studies, we used a three-step approach to determine copy number. We used read counts over the whole glycophorin region to detect samples with duplications (more than expected number of reads) and deletions (fewer than expected number of reads). We then used window-based analysis of sequence read depth and paralogue-specific allele-specific PCR and Sanger sequencing to refine copy number breakpoints. Finally, we validated the structure of selected variants using fibre-FISH. Our approach has the advantage that it does not rely on a sudden change in sequence read depth for CNV detection by a HMM, which may be compromised by poor mappability of some sequence reads in the breakpoint region and assumptions about the absence of somatic variation, with the consequence that the expected copy number reflecting an integer value. However, our approach cannot detect smaller copy number changes, with an estimated threshold of ~ 60 kb for heterozygous changes of and ~ 30 kb for homozygous changes. This is because, for these sizes, the relative increase or decrease in the number of mapped reads at the glycophorin region is likely to be below the threshold used to call a copy number change. We also make assumptions that each variant is a simple deletion or duplication with one breakpoint, unless clearly shown to be otherwise by fibre-FISH, such as for DUP5. Long read DNA sequencing will help to clarify the variation in this region further and will be able to resolve the extent to which our assumptions have been valid.

Previous work has shown that the DUP4 variant carried by the sample HG02554 shows somatic mosaicism, leading to the suggestion that somatic mosaicism may be a feature of glycophorin structural variants [12] . In this study, our fibre-FISH analyses identified no other potential somatic variants at the glycophorin locus, showing that it is not a common feature of 1000 Genomes lymphoblastoid cell lines nor of non-DUP4 variants. This suggests that somatic mosaicism is either restricted to DUP4 variants in general or restricted to the particular DUP4 sample HG02554, although a more thorough investigation of high coverage genome sequences will be needed to address this issue.

## Conclusion

We identify nine new structural variants at the human glycophorin locus, characterise breakpoints and mutational mechanisms for known and novel structural variants, and show that recombination hotspot activity has influenced the nature of the structural variants observed. For some of the variants, targeted high coverage sequence using very long reads will help refine some of the breakpoints. Further efforts are needed to characterise the phenotypic effects of particular variants involving gain, loss and fusion of glycophorin genes.

## Methods

### Samples analysed in this study

For this study we analysed whole genome sequences from 2492 individuals from the 1000 Genomes Project, 391 individuals from the Gambian Genomes project, 274 individuals from the Simons Diversity Project, and 1325 individuals from the Brazilian Genomes project. Of these, the 1000 Genomes project samples analysed here had been previously analysed specifically for glycophorin copy number variation using different approaches [11].

### Sequencing data

Sequence alignment files (.bam format) from four cohorts (1000 Genomes Project ENA accession number PRJNA262923) with a mean coverage of 7.4x [32], Simons Diversity Project ENA accession number PRJEB9586 with a mean coverage of 43x [33], and the Gambian Genome Diversity project mean coverage 4x, ENA study IDs ERP001420, ERP001781, ERP002150, ERP002385) [34] were downloaded from the European Nucleotide Archive or from the International Genome Sample Resource site http://www.internationalgenome.org/data-portal/ [35]. We also analysed Brazilian sequence alignment files from the SABE (Health, Wellbeing and Aging) study [36] and a sample of cognitively healthy octogenarians enrolled at the Human Genome and Stem Cell Research Center (80+), with a mean coverage of 30x for 1325 individuals generated at Human Longevity Inc. (HLI, San Diego, California) [37].

DNA sequences from the 1000 Genomes project and the Simons diversity project had been previously aligned to reference GRCh37 (hg19) to generate the alignment bam files. The exception is sample NA18605, which was previously sequenced at high coverage [38] downloaded as paired-end Illumina sequences in fastq format (ENA sample accession number SAMN00001619), and aligned to GRCh37 using standard approaches: FastQC v0.11.5 and Cutadapt v01.11 to trim reads and adapters, mapping using BWA-MEM v0.7.15, processing of the BAM files using SAMtools v1.8, local realignment was done using GATK v3.6 and duplicate reads marked using Picard v.1 and removed using SAMtools. Samples from the Brazilian genomes and the Gambian genome diversity project had been aligned to GRCh38.

Throughout this paper, all loci are given using GRCh37 reference genome coordinates. For analyses on GRCh38 alignments, genome coordinates were translated from the GRCh37 coordinates using the Liftover tool within the UCSC Genome Browser [39].

### Structural variant detection

For each sample, we used SAMtools (SAMtools view –c –F 4) [24] on indexed bam files to count mapped reads to the glycophorin region (chr4:144745739–145,069,133) and a reference region chr4:145516270–145,842,585. The reference region has no segmental duplications, and is absent from copy number variation according to the gold standard track of the database of Genomic Variants (DGV) [40]. A ratio of the number or reads mapping to the glycophorin region to the number of reads mapping to the reference region allows an estimate of the total increase or decrease of sequence depth spanning the glycophorin region (reflecting copy number gain or copy number loss, respectively). Because the size of the regions used for sequence read count is ~ 320 kb, and spans the whole glycophorin region, we would not expect copy number losses within the region to necessarily show read depth ratios of 0 or 0.5 for homozygous or heterozygous losses respectively, unless the whole 320 kb region is deleted. For similar reasons we would not expect homozygous or heterozygous copy number gains to show values of 1.5 or 2. Following plotting these data for each cohort on a histogram, observation of distinct clusters (supplementary Fig. 1) allowed us to identify samples with a ratio below 0.9 as potential copy number losses and those above 1.1 as potential copy number gains. The main peak of the histogram below 0.9 is at ~ 0.8, and above 1.1 is at 1.2, suggesting that the copy number gains or losses identified in those peaks in the histograms are ~ 100 kb and heterozygous. Samples showing rations of ~ 0.6 for losses or ~ 1.4 for gains represent either larger copy number changes in the heterozygous state, or homozygous ~ 100 kb copy number alterations.

For the samples with potential copy number gains and losses, the mapped reads were counted across the glycophorin region in 5 kb non-overlapping windows, normalised to the average read count across the whole region, then normalised to diploid copy number. The resulting values were plotted across the genomic region. The presence and nature of structural variants were assessed by examination of quality of the plots, ensuring that copy number gains and losses and a consistent gain or loss of sequence read depth across a contiguous region. Variants were grouped and called as the same variant by plotting together with a reference sample for that variant. For the 1000 Genomes project, 6 samples were identified as harbouring copy number gains or losses across the glycophorin region, but failed to pass this subsequent 5 kb window step because sequence read depth was noisy across the region and no consistent region showing loss or gain of read depth was seen. For the Simons Diversity Project samples, 114 potential deletions were identified, much more than in other cohorts (Supplementary Fig. 1). Inspection of these plots showed that 101 of these samples showed a high level of sequence depth ratio noise, and a small apparent ~ 15 kb deletion at the *GYPE* gene. This deletion was not found previously by others [11] by us in any other cohort, and coincides with a region of low mappability, suggesting that this may be an artefact caused either by particular filtering conditions or the particular genome assembly (GRCh37d5) that includes decoy sequences. These 101 samples were treated as being homozygous for the reference structure.

Twenty samples had been sequenced both by the 1000 Genomes project and the Simons’ Diversity Project. Our copy number calls were identical between both replicate genome sequences across all 20 samples, with three samples showing a copy number variant. The twenty samples included samples that showed the putative 15 kb deletion in the Simons diversity samples, but not in the 1000 Genomes samples, further supporting our assertion that this was an artefact.

### Fibre-FISH

The probes used in this study included four WIBR-2 fosmid clones selected from the UCSC Genome Browser GRCh37/hg19 assembly and a 3632-bp PCR product that is specific for the glycophorin E repeat [12] . Probes were made by amplification with GenomePlex Whole Genome Amplification Kits (Sigma-Aldrich) as described previously [33] . Briefly, the purified fosmid DNA and the PCR product were amplified and then labeled as follow: G248P86579F1, G248P89366H1 and glycophorin E repeat-specific PCR product were labelled with digoxigenin-11-dUTP, G248P8211G10 was labelled with biotin-16-dUTP, G248P85804F12 was labeled with DNP-11-dUTP and G248P80757F7 was labeled with Cy5-dUTP. All labeled dUTPs were purchased from Jena Bioscience.

The preparation of single-molecule DNA fibers by molecular combing and fiber-FISH was as previously published [33], with the exception of post-hybridization washes, which consisted of three 5 min washes in 2× SSC at 42 °C, instead of two 20 min washes in 50% formamide/50% 2× SSC at room temperature.

### Breakpoint analysis using PCR and sanger sequencing

Using the 5 kb window sequence read count data, PCR primers were designed so that a PCR product spanned the predicted breakpoints for each deletion and duplication. The 3′ nucleotide for each PCR primer was designed to match uniquely to a particular glycophorin repeat, and to mismatch the other two glycophorin repeats. Annealing specificity of the PCR primer was enhanced by incorporating a locked nucleic acid at that particular 3′ position of the PCR primer [21]. Long-range PCR amplification used 10 ng genomic DNA in a final volume of 25.5 μl, including 0.5 μl of each 10 μM primer, 0.075 U *Pfu* DNA polymerase, 0.625 U *Taq* DNA polymerase, and 2.25 μl of PCR buffer (45 mM Tris-HCl (pH 8.8), 11 mM ammonium sulphate, 4.5 mM magnesium chloride, 6.7 mM 2-mercaptoethanol, 4.4 mM EDTA (pH 8.0), 113 μg/mL non-acetylated Bovine Serum Albumin (BSA) (Ambion®) and 1 mM of each dNTP (Promega) [41]). The reaction was thermal cycled as follows: 94 °C 1 min, followed by 20 cycles of 94 °C for 15 s, x°C for 10 min, followed by 15 cycles of 94 °C for 15 s, x°C for 10 min + 15 s for each successive cycle, followed by a final extension at 72 °C for 10 min, where x is the annealing temperature for a particular primer pair shown in supplementary Table 1. PCR products were purified using agarose gel electrophoresis [42] and Sanger sequenced using standard approaches. PCR primers are shown in supplementary Table 1. Multiple alignments with paralogous reference sequences used MAFFT v7 [43] available at the EMBL-EBI Job Dispatcher framework [44]. A breakpoint was called in the transition region between three paralogous sequence variants corresponding to one glycophorin repeat and three paralogous sequence variants corresponding to the alternative glycophorin repeat.

### Breakpoint analysis using high depth sequences

For particular variants, copy number breakpoints were refined by inspecting sequence read depth in 1 kb windows spanning the likely breakpoints identified by the 5 kb window analysis. Changes in read depth were then confirmed directly using the Integrative Genome Viewer [45].

### Nomenclature of variants

We used the same nomenclature as reference [11] when our variant could be identified as the same variant in the same sample from the 1000 Genomes project. In some instances, we could not distinguish particular singleton variants called from more common called variants. For example, DUP27 carried by sample NA12249 could not easily be distinguished from the more frequent DUP2, and DUP24 carried by HG04038 could not be distinguished from DUP8. Other variants, which either had not been unambiguously identified in the 1000 Genomes previously or were identified in other sample cohorts, were given DEL or DUP numbers following on from variants catalogued previously. Variant data are available on dbVar https://www.ncbi.nlm.nih.gov/dbvar/ accession number nstd177. A list of the samples carrying particular variants is also included as supplementary data.

### Analysis of recombination hotspots

Previously published data on hotspot location and type [25] was converted to BED format and intersected with the breakpoint locations in BED format using BEDTools v 2.28.0 [46]. The statistical significance of the overlap was calculated using the fisher command in BEDTools, which uses a Fisher’s exact test on the number of overlaps observed between two BED files.

## Supplementary information

**Additional file 1.** Supplementary Table 1

**Additional file 2: Supplementary Fig. 1.** Histograms of sequence read depths of the glycophorin region. Histograms of normalised sequence read depths of the four cohorts used for this study, with red indicating putative deletions and blue putative duplications. The Brazilian Genomes samples are new to this study, all other samples have publicly available genome sequence. a) 1000 Genomes Project (2492 individuals) Gambian Genome Variation Project (391 individuals). b) Simons Diversity Project (274 individuals). c) Brazilian Genomes Project (1325 individuals)

**Additional file 3: Supplementary Fig. 2.** Sequence alignment of DUP2 and DUP27 variants across their breakpoints. The figure shows an alignment of the DUP2 variant sequence and the DUP27 variant sequence from the index samples NA18593 and NA12249 respectively. Also aligned are the reference GYPB and GYPA sequences. Variable nucleotides in the alignment are coloured depending on whether they are the same as GYPA in that position (purple) or GYPB in that position (orange). Red arrows indicate differences between the two variants.

**Additional file 4: Supplementary Fig. 3.** Summary of the positions of deletion and duplication variants in this study. The complex DUP5 rearranged variant is not shown.

**Additional file 5.** Supplementary data.

## Data Availability

Variant data is available on dbVar https://www.ncbi.nlm.nih.gov/dbvar/ accession number nstd177. Genome sequence data for the 1000 Genomes project samples and Gambian Genome Variation Project samples are available at https://www.internationalgenome.org/data. Brazilian genome data corresponding to the region analysed in this paper are available on request.

## Abbreviations

CNV: Copy number variation
FISH: Fluorescent in-situ hybridisation
GWAS: Genomewide association study
HMM: Hidden Markov Model
PCR: Polymerase chain reaction
