# Supplementary material for: Structural variation of the malaria-associated human glycophorin A-B-E region
Source: BMC Genomics. 2020 Jun 29;21:446. doi: 10.1186/s12864-020-06849-8 (PMC7325229; doi:10.1186/s12864-020-06849-8)
Supplement: Supplementary file 1 — Additional file 1. Supplementary Table 1 [file 12864_2020_6849_MOESM1_ESM.docx]

**Supplementary table 1**

| **Variant** | **Primer name** | **Primer Sequences 5′ - 3′** | **Annealing temperature °C** |
| --- | --- | --- | --- |
| DEL1 | *GYP*_DEL1_F | CCAGTTGCCTCTAAGTCCAT[C] | 65 |
|  | *GYP*_DEL1_R | GCAGTGCACACCCTGG[A] |  |
| DEL2 | *GYP*_DEL2_F | AGGCAAAAGCTGAGGTCTT[C] | 65 |
|  | *GYP*_DEL2_R | CAGCCTCTGGTAACCACTGTTA[C] |  |
| DEL6 | *GYP*_DEL6_F | GAAGAAAGAGCTAATTCCAT[G] | 63 |
|  | *GYP*_DEL6_R | AGTTGGAACTTGCAAACTTA[G] |  |
| DEL7 | *GYP*_DEL7_F | ATCCTGCACTAGAAATTCCTCCCA[C] | 65 |
|  | *GYP*_DEL7_R | GATCAGAAAAGCAAAATGGGGC[A] |  |
| DEL13 | *GYP_*DEL13_F | CCCTCACCCACAGAAAGAAC[C] | 62 |
|  | *GYP_*DEL13_R | GGAAGGTTTTAGAAGTCTTCAGTTG[G] |  |
| DUP2 | *GYP*_DUP2_F | CAGAGAAATGATGGGCAAGTTGT[A] | 62 |
|  | *GYP*_DUP2_R | ACTGCGTGGACATAGAGCGTAT[T] |  |
| DUP3 | *GYP*_DUP3_F | CAAATGAAGTCAAACATCTTC[A] | 63.5 |
|  | *GYP*_DUP3_R | CTTGAGACACTCCTTTATATGCTA[C] |  |
| DUP5 | *GYP*_DUP5_F | AGCTTGGATGAGATAAATGTCC[T] | 65 |
|  | *GYP*_DUP5_R | ATTGGATTCTGATGTGCGG[C] |  |
| DUP14 | *GYP*_DUP14_F | GTCTTTAAAGTATTGTTTCGTGC[A] | 65 |
|  | *GYP*_DUP14_R | AGGTTAATCTAAAACTTTAGAGCAA[C] |  |
| DUP29 | *GYP*_DUP29_F | GCTGCCAGATCAATAGC[G] | 64 |
|  | *GYP*_DUP29_R | TAGTAGTATAAACCACAGTGCCTC[A] |  |

These primer pairs have been designed to specifically amplify across the breakpoint of the variant shown in the index samples for each variant shown in table 1. They can be used to test for the presence of these variants in genomic DNA. Nucleotides that are linked nucleic acids are shown in square brackets.
