# Supplementary figures and images for: Structural variation of the malaria-associated human glycophorin A-B-E region
Source: BMC Genomics. 2020 Jun 29;21:446. doi: 10.1186/s12864-020-06849-8 (PMC7325229; doi:10.1186/s12864-020-06849-8)

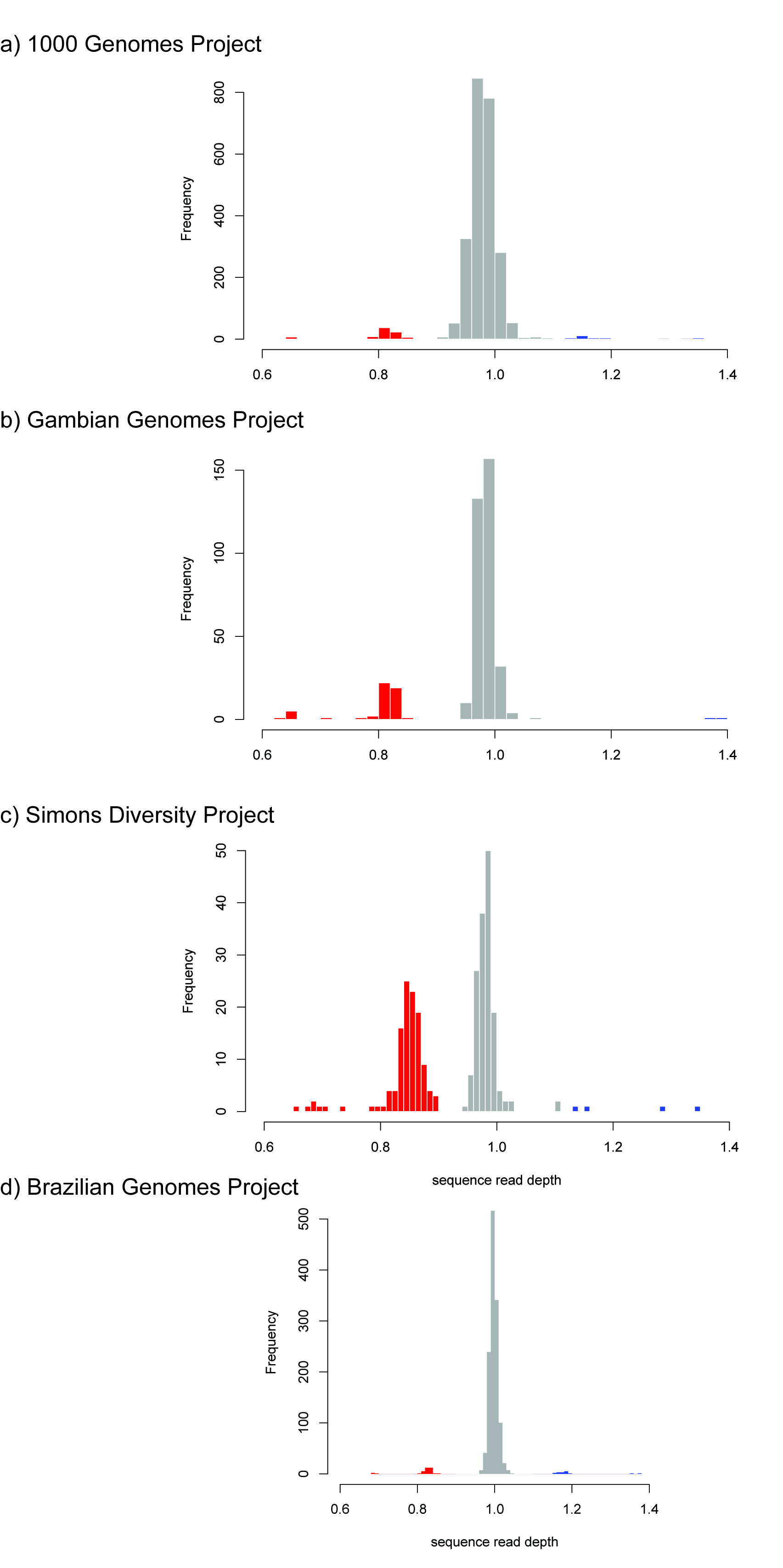

Supplement: Supplementary file 2 — Additional file 2: Supplementary Fig. 1. Histograms of sequence read depths of the glycophorin region. Histograms of normalised sequence read depths of the four cohorts used for this study, with red indicating putative deletions and blue putative duplications. The Brazilian Genomes samples are new to this study, all other samples have publicly available genome sequence. a) 1000 Genomes Project (2492 individuals) Gambian Genome Variation Project (391 individuals). b) Simons Diversity Project (274 individuals). c) Brazilian Genomes Project (1325 individuals) [file 12864_2020_6849_MOESM2_ESM.tif]

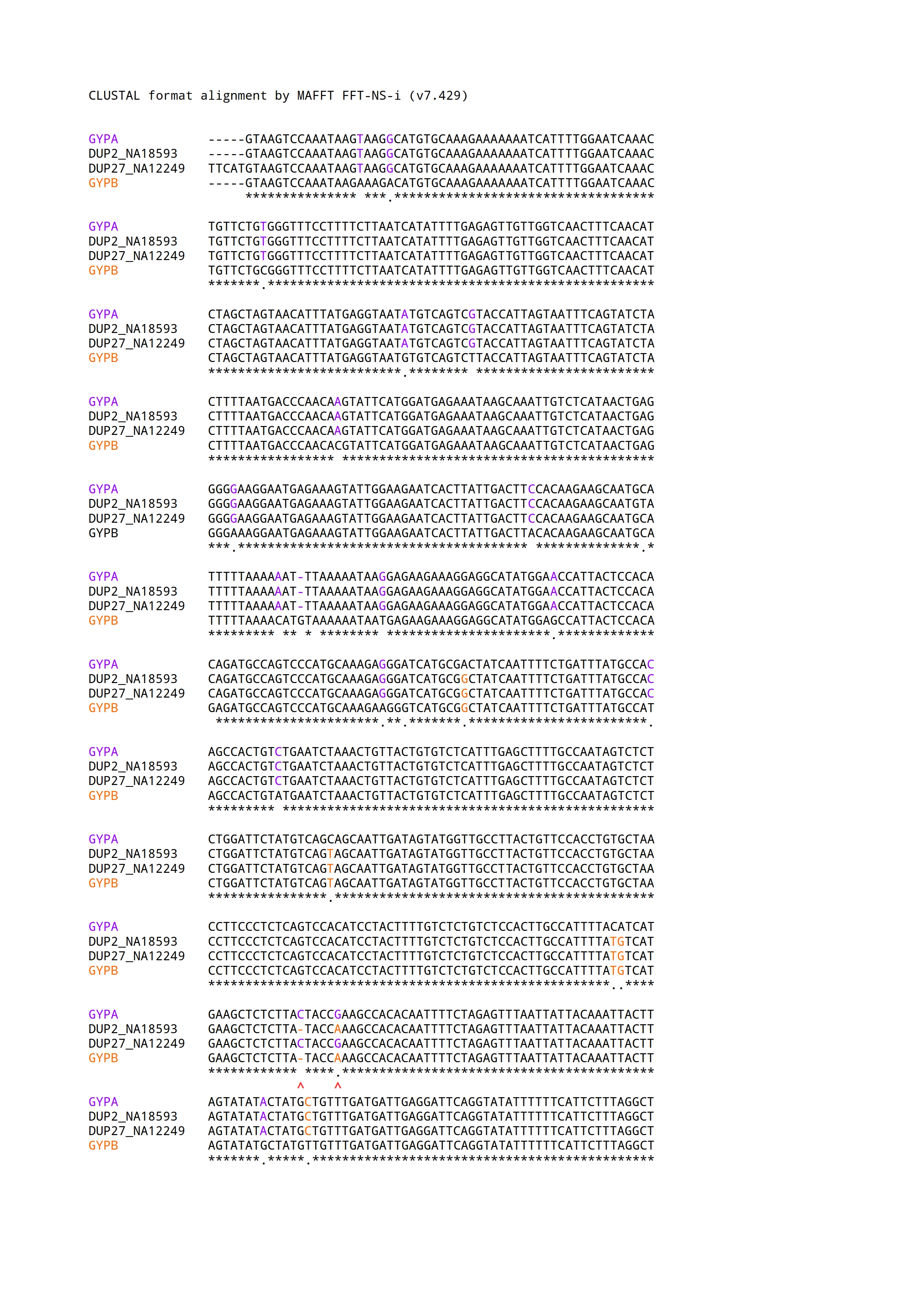

Supplement: Supplementary file 3 — Additional file 3: Supplementary Fig. 2. Sequence alignment of DUP2 and DUP27 variants across their breakpoints. The figure shows an alignment of the DUP2 variant sequence and the DUP27 variant sequence from the index samples NA18593 and NA12249 respectively. Also aligned are the reference GYPB and GYPA sequences. Variable nucleotides in the alignment are coloured depending on whether they are the same as GYPA in that position (purple) or GYPB in that position (orange). Red arrows indicate differences between the two variants. [file 12864_2020_6849_MOESM3_ESM.jpg]

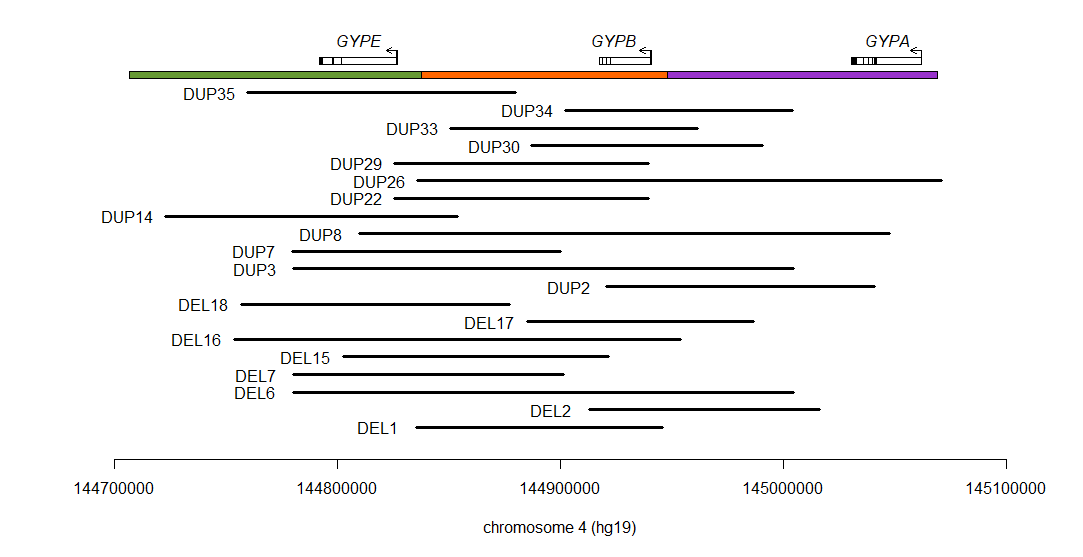

Supplement: Supplementary file 4 — Additional file 4: Supplementary Fig. 3. Summary of the positions of deletion and duplication variants in this study. The complex DUP5 rearranged variant is not shown. [file 12864_2020_6849_MOESM4_ESM.tiff]
